# Adaptive strategy of allohexaploid wheat to long-term salinity stress

**DOI:** 10.1186/s12870-020-02423-2

**Published:** 2020-05-12

**Authors:** Nadeem Bhanbhro, Binbin Xiao, Lei Han, Huiying Lu, Huan Wang, Chunwu Yang

**Affiliations:** 1 Key laboratory of Molecular Epigenetics of Ministry of Education (MOE), Northeast Normal University, Changchun, 130024 China; 2 Department of Agronomy, Jilin Agricultural University, Changchun, 130118 China

**Keywords:** Wheat, Salinity stress, Energy partitioning, ABA, GA

## Abstract

**Background:**

Most studies of crop salinity tolerance are conducted under short-term stress condition within one growth stage. Understanding of the mechanisms of crop response to long-term salinity stress (LSS) is valuable for achieving the improvement of crop salinity tolerance. In the current study, we exposed allohexaploid wheat seeds to LSS conditions from germination stage to young seedling stage for 30 days. To elucidate the adaptive strategy of allohexaploid wheat to LSS, we analyzed chloroplast ultrastructure, leaf anatomy, transcriptomic profiling and concentrations of plant hormones and organic compatible solutes, comparing stressed and control plants.

**Results:**

Transcriptomic profiling and biochemical analysis showed that energy partitioning between general metabolism maintenance and stress response may be crucial for survival of allohexaploid wheat under LSS. Under LSS, wheat appeared to shift energy from general maintenance to stress response through stimulating the abscisic acid (ABA) pathway and suppressing gibberellin and jasmonic acid pathways in the leaf. We further distinguished the expression status of the A, B, and D homeologs of any gene triad, and also surveyed the effects of LSS on homeolog expression bias for salinity-tolerant triads. We found that LSS had similar effects on expression of the three homeologs for most salinity-tolerant triads. However, in some of these triads, LSS induced different effects on the expression of the three homeologs.

**Conclusions:**

The shift of the energy from general maintenance to stress response may be important for wheat LSS tolerance. LSS influences homeolog expression bias of salinity-tolerant triads.

## Background

The genome of allohexaploid common wheat (*Triticum aestivum* L., genome BBAADD) was generated through two allopolyploidization events. The first such event resulted in the formation of allotetraploid wheat (*Triticum turgidum*, genome BBAA) 0.36–0.5 million years ago [1–3]. About 8500 years ago, the second allopolyploidization event (hexaploidization) produced allohexaploid wheat. The second hexaploidization occurred naturally by hybridization between a domesticated allotetraploid wheat (genome BBAA) and a diploid species *Aegilops tauschii* (genome DD) and subsequent chromosome doubling [1–3]. The BBAA and DD genomes have co-existed and interacted in allohexaploid wheat cells for only 8500 years, during which time the extensive expression and functional partitioning of homeologs occurred, in addition to alterations in chromosome structure [4–6]. Due largely to the interactions between the DD and BBAA genomes, the multiple and complex BBAADD genome confers on allohexaploid wheat increased physiological and ecological plasticities that contribute to its remarkable tolerance to diverse stress conditions [1, 7–9]. Wheat salinity tolerance was increased immediately following hexaploidization, with the allohexaploid exhibiting greater salt tolerance than its tetraploid progenitor, *T. turgidum* [8, 10]. It is unclear as to whether salinity stress can alter the expression patterns of the A, B and D homeologs within a gene triad, and whether the expression pattern change of homeologs is associated with the salinity stress response of allohexaploid wheat.

The ultimate aim of research into salinity tolerance is the improvement of salinity tolerance in crop plants and the subsequent utilization of extensively salinized lands. Unfortunately, most investigations of crop salinity tolerance are conducted in a greenhouse under short-term stress condition within a single growth stage. If crop plants are grown in naturally saline farmland, the growth of crop plants will be subjected to soil salinity stress for their entire life cycle, across different growth and development stages. Hence, an understanding of the mechanisms of crop response to long-term salinity stress (LSS) is helpful for crop salinity tolerance improvement. To our knowledge, the mechanisms by which plants respond to salinity stress have been largely investigated using the short-term salinity stress system, whereas LSS conditions are rarely used to investigate salinity tolerance [11–13]. LSS may have more complex effects on plant metabolism and development than short-term salinity stress. The response of plants to LSS may be a more complex process, involving almost all metabolic processes occurring at different hierarchical levels, including molecular, sub-cellular, cellular, tissue and whole-plant levels and across different growth stages. To our knowledge, little is known of the coordinated response of crop plants to LSS. We thereby subjected allohexaploid wheat seeds to LSS conditions from germination stage to young seedling stage for 30 days. We analyzed chloroplast ultrastructure, leaf anatomy, concentrations of plant hormones, 37 organic compatible solutes, two inorganic ions, and transcriptomic profiling of the stressed plants relative to control unstressed plants. We also attempted to associate transcriptomic response with the comprehensive physiological response. The aims of the research were to describe the physiological response of allohexaploid wheat plants to LSS across the germination and seedling stages, to identify the key response genes of the wheat plants under LSS, to demonstrate how LSS affected the expression patterns of homeologs, and to elucidate gene expression and physiological adaptation strategies to LSS.

## Results

### Anatomy and solutes

**Fig. 1:**
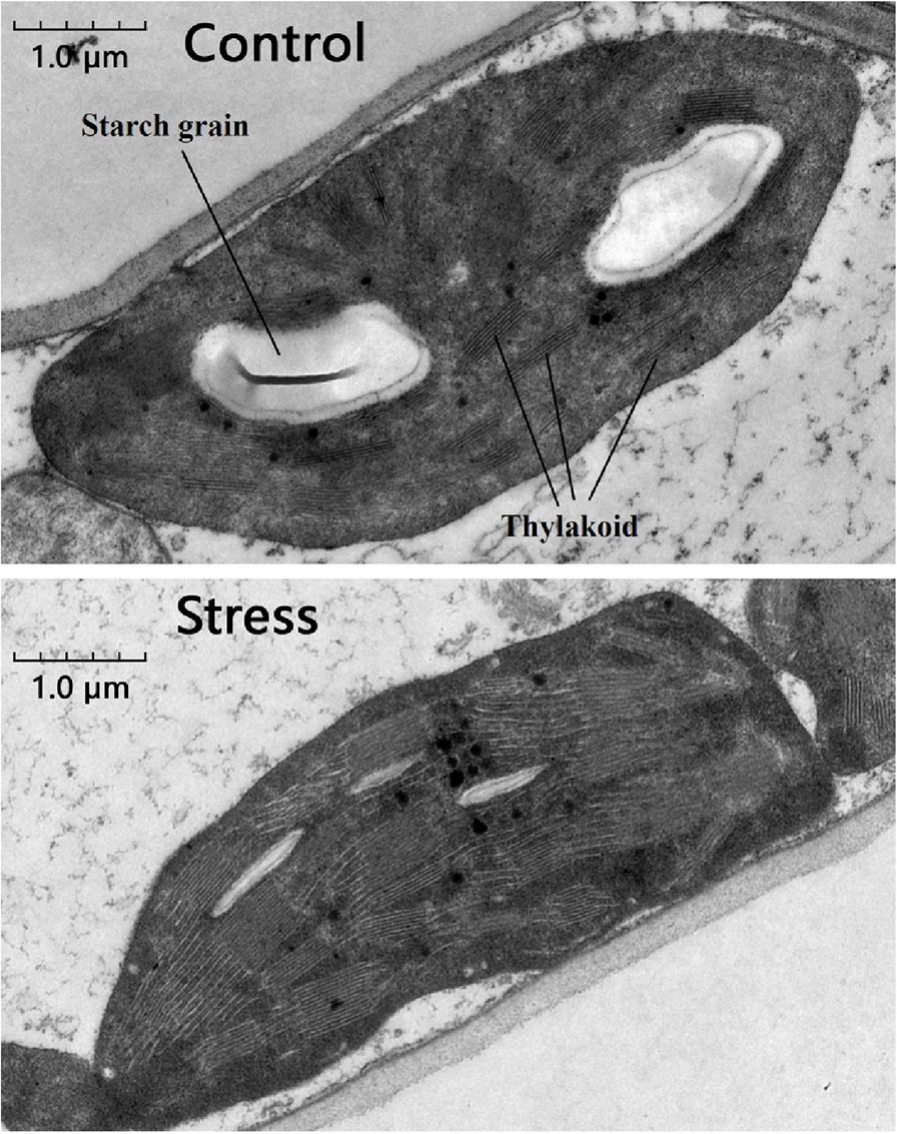
Effects of long-term salinity stress on chloroplast ultrastructure in allohexaploid wheat. The wheat seeds were treated with 100 mM NaCl for 30 days

**Fig. 2:**
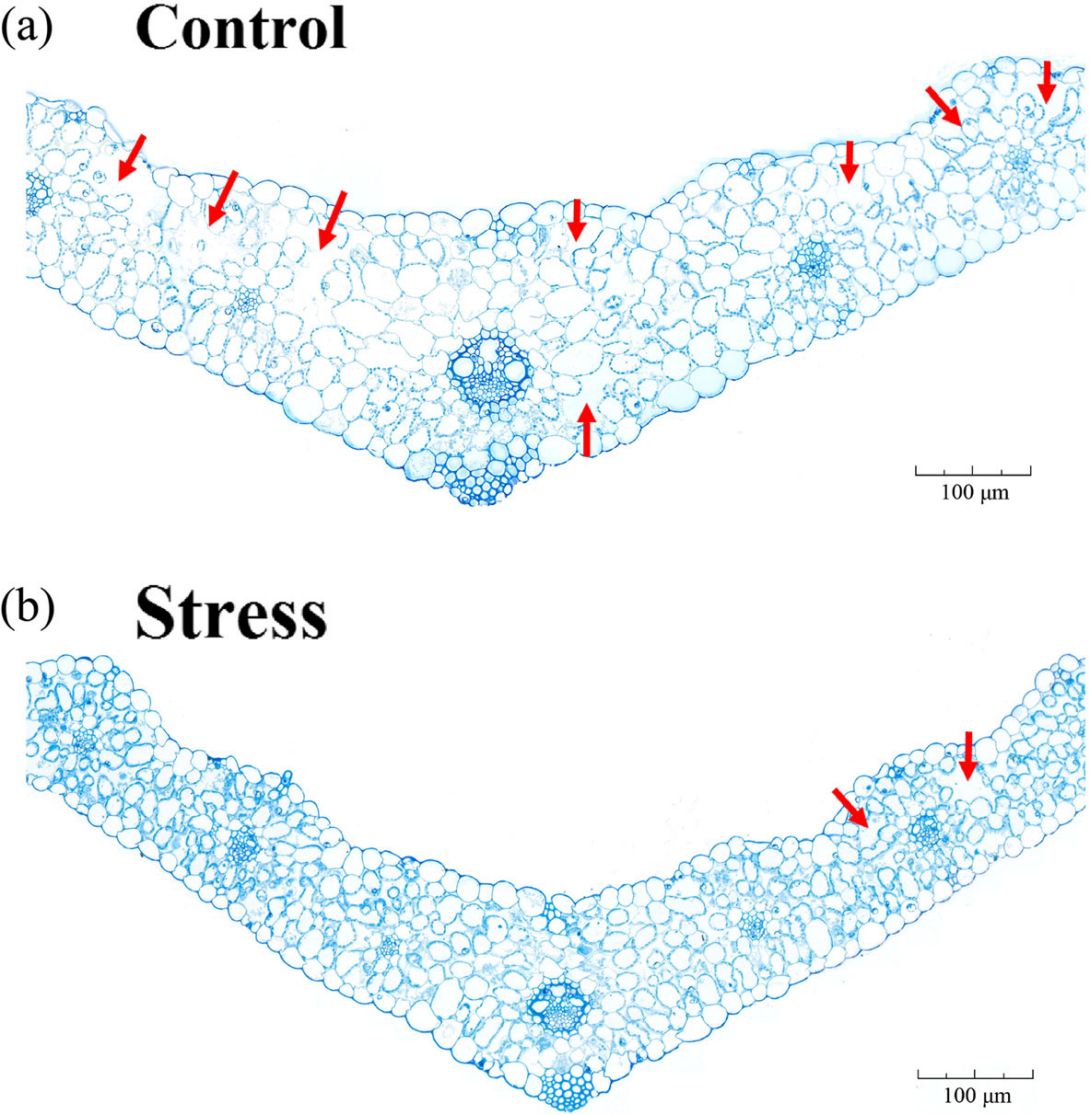
Effects of long-term salinity stress on leaf anatomy in allohexaploid wheat. The wheat seeds were treated with 100 mM NaCl for 30 days. Red arrow shows aerenchyma or stomatic chamber

**Fig. 3:**
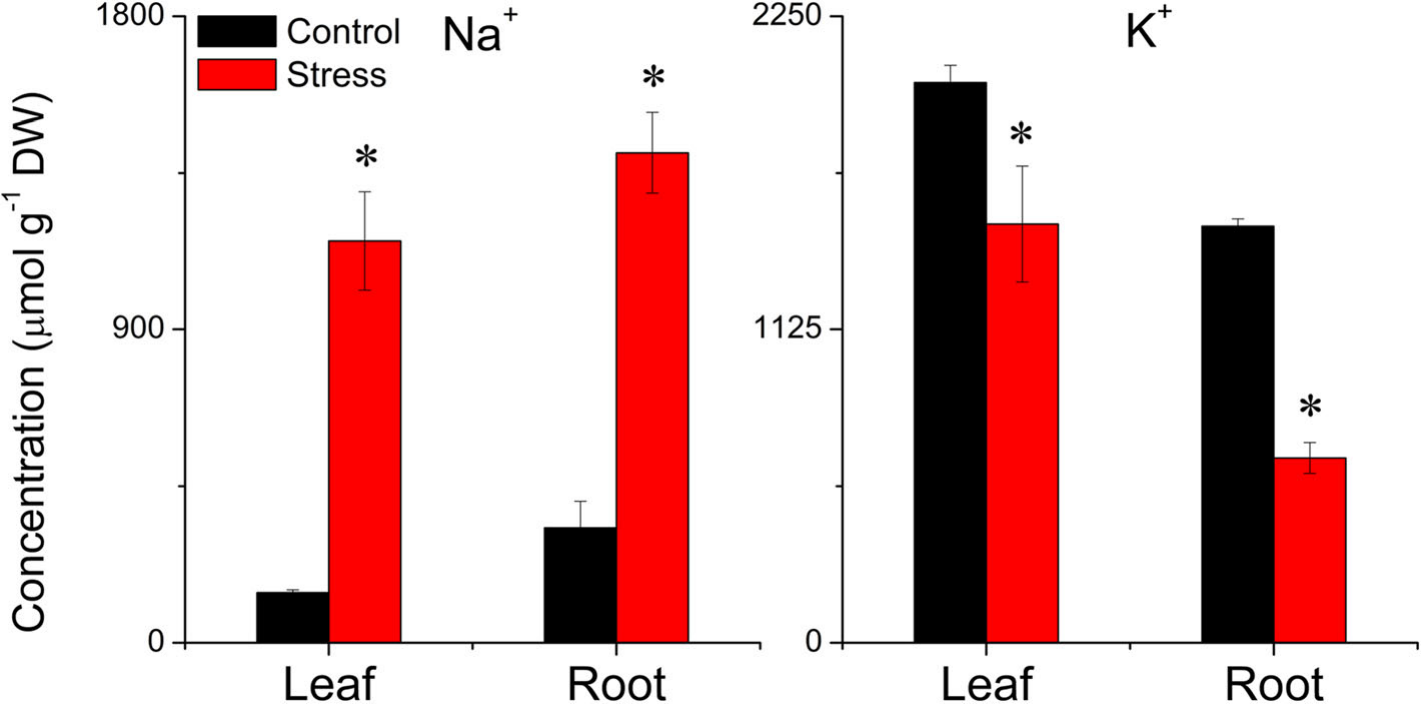
Effects of long-term salinity stress on Na^+^ and K^+^ contents in allohexaploid wheat. The wheat seeds were treated with 100 mM NaCl for 30 days. Values are means (± standard deviation) of three biological replicates. Statistical significance between control and stress treatments was determined by *t*-test, and marked as * (*P* < 0.05)

We compared the anatomy of leaves and the ultrastructure of chloroplasts in salt-stressed and control wheat. We found that the thylakoids in chloroplasts of stressed plants had a higher packing density than those of control plants, with more and larger starch grains in control plants than in stressed plants (Fig. 1). Anatomical comparisons showed that control plants had more and larger aerenchyma than stressed plants (Fig. 2). LSS significantly increased the Na^+^ concentration and decreased the K^+^ concentration in both leaves and roots (Fig. 3). To determine the contribution of each solute toward osmotic adjustment, we calculated the percentage contribution of the molarity of each solute to total molarity. A change of 50% in the stressed, relative to the control plants, and a *P* value of < 0.05 were considered to reflect a significant change, with the concentrations of serine, asparagine, histidine, and proline being significantly up-regulated in the leaves under LSS, whereas the concentration of proline was significantly upregulated only in the roots of stressed plants (Additional file 2: Table S1). The concentrations of fucose, lactose and maltose were significantly increased in the leaves under LSS, whereas the concentrations of almost all carbohydrates were increased in the stressed roots. In stressed leaves, alanine (10.15%), proline, (8.80%), sucrose (44.6%) and maltose (9.69%) made much greater contributions to total molarity than did other solutes, and were considered to be the major compatible osmotic regulators (Additional file 2: Table S1). In stressed roots, alanine (12.79%), glucose (8.72%), sucrose (19.36%) and fructose (17.55%) made much greater contributions to total molarity than did other solutes (Additional file 2: Table S1).

### Plant hormones

**Fig. 4:**
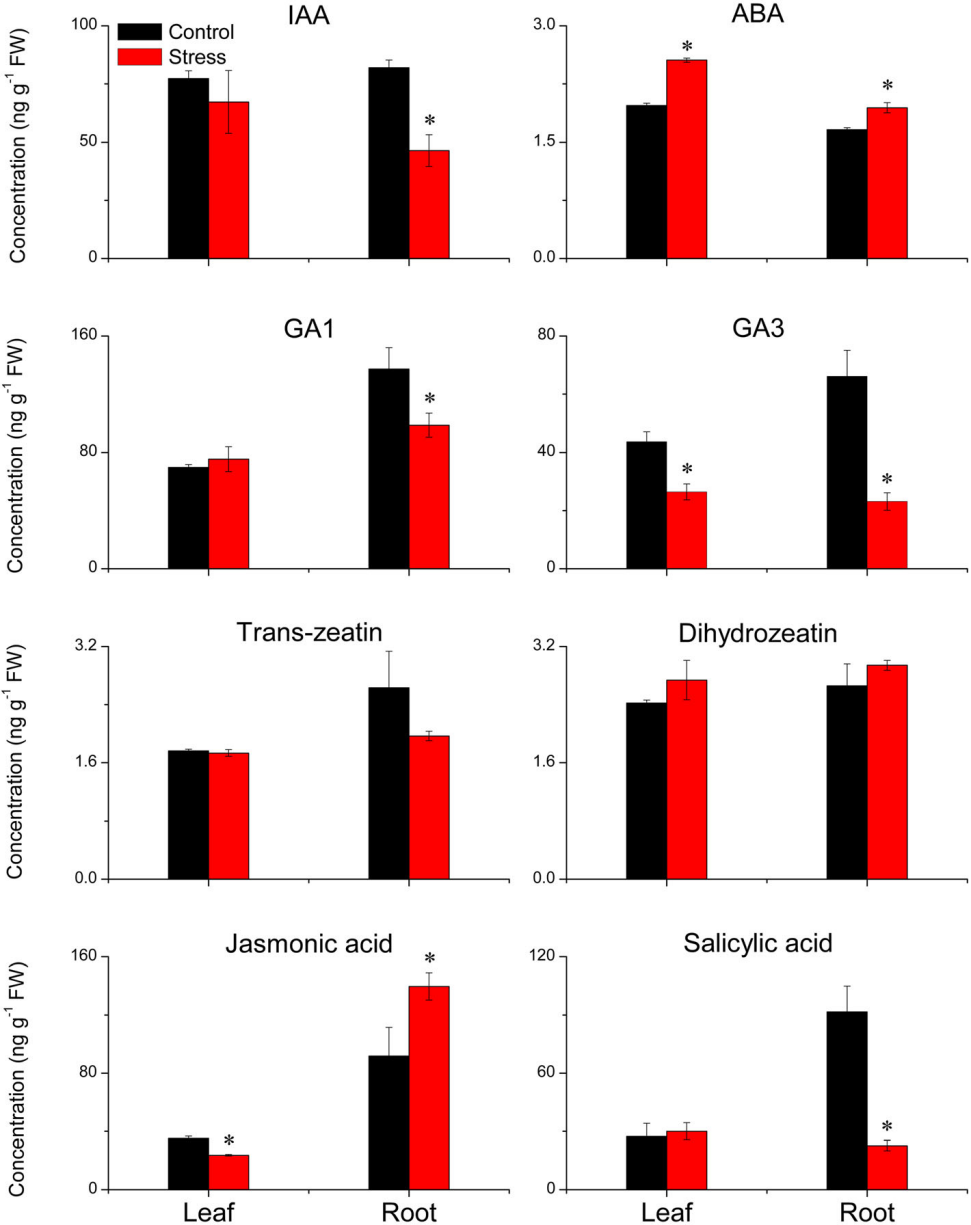
Effects of long-term salinity stress on plant hormone concentrations in allohexaploid wheat. The wheat seeds were treated with 100 mM NaCl for 30 days. Values are means (± standard deviation) of three biological replicates. Statistical significance between control and stress treatments was determined by *t*-test, and marked as * (*P* < 0.05)

The contents of jasmonic acid (JA), dihydrozeatin, *trans*-zeatin, abscisic acid (ABA), salicylic acid (SA), gibberellin A1 (GA_1_), indole-3-acetic acid (IAA), and gibberellin A3 (GA_3_) were determined (Fig. 4). LSS did not significantly affect the concentrations of dihydrozeatin and *trans*-zeatin, but significantly increased the JA concentration in the roots, and decreased its concentration in the leaves. LSS increased ABA concentration in the leaves, and enhanced its concentration in the roots. LSS decreased GA_3_ concentrations in both leaves and roots, and reduced the concentrations of IAA, GA_1_ and SA in the roots, but did not significantly affect their concentration in the leaves (Fig. 4).

### Transcriptomic profiling

In transcriptomic profiling, 9311 genes (3741 up-regulated differentially expressed genes (DEGs) and 5570 down-regulated DEGs) and 6572 genes (4896 up-regulated DEGs and 1676 down-regulated DEGs) were differentially expressed in the roots and leaves, respectively. We subjected all the DEGs (adjusted *P* value< 0.05 and |log2fold change| ≥ 1) to KEGG enrichment (Additional file 1: Figure S1–S2). The leaf DEGs were significantly enriched with respect to 30 pathways (adjusted *P* value< 0.05), and the root DEGs were enriched with respect to 13 pathways (adjusted *P* value< 0.05). Four enriched pathways common to both roots and leaves were plant hormone signal transduction, alpha-linolenic acid metabolism, glutathione metabolism and linoleic acid metabolism (Additional file 1: Figure S1–S2). In the leaves, almost all DEGs were up-regulated in five pathways, namely cutin, suberin and wax biosynthesis, fatty acid elongation, biosynthesis of unsaturated fatty acids, fatty acid metabolism, and flavonoid biosynthesis (Additional file 1: Figure S1).

**Fig. 5:**
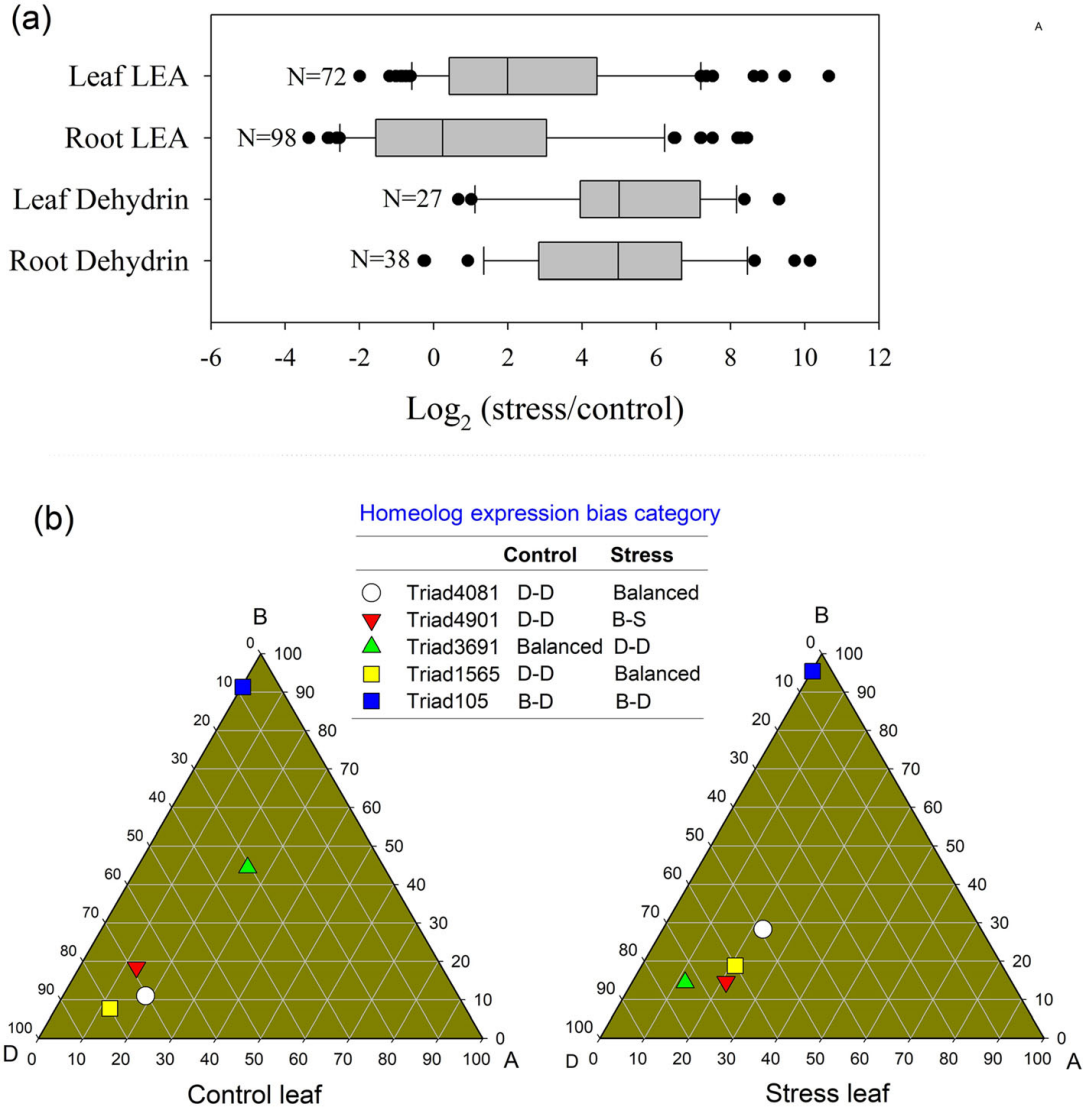
Effects of long-term salinity stress on expression of *LEA* and *dehydrin* genes. **a** Box plot displaying fold changes for all differentially expressed *late embryogenesis abundant* (*LEA*) and *dehydrin* genes. **b** Ternary plot displaying homeologous expression bias categories of five typical *LEA* gene triads. The wheat seeds were treated with 100 mM NaCl for 30 days. The three vertices (A, B and D) of the triangle represent ideally dominant expression of the corresponding A, B and D homeologs, respectively. B-S, B suppressed; B-D, B dominant; D-D, D dominant

Nine *ABF* (ABA-associated transcription factor) genes were greatly up-regulated in the leaf in response to LSS (Table 1). Two GA-related *DELLA* genes and ten JA-related *JAZ* genes were highly up-regulated in wheat leaves under LSS (Table 1). All DEGs (six *FAR*, six *MAH1* and seven *WSD1* genes) related to wax biosynthesis were up-regulated in the leaf under LSS (Additional file 1: Figure S1 and S3). The *TaHKT1;5D* gene (TraesCS4D01G361300) was highly up-regulated in roots, but not in leaves, in response to LSS (Additional file 2: Table S2). The expression of four *NHX* genes, four *HKT* genes, two *V-type H*^*+*^*ATPase* genes, one *potassium channel SKOR* gene and three *potassium transporter* genes, was greatly up-regulated in roots under LSS. One *SOS1* gene was also up-regulated in the root under LSS (Additional file 2: Table S2). Two aquaporin *TIP2;3* genes were greatly up-regulated in leaves, but not in roots of stressed plants (Additional file 2: Table S2). In addition, we observed that 69 *late embryogenesis abundant* (*LEA*) genes and 39 *dehydrin* genes were significantly up-regulated in either roots or leaves under LSS (Additional file 2: Table S2 and Fig. 5). Under LSS, many genes showed greater than 300-fold up-regulation, including two *LEA* genes (TraesCS1B01G381400 and TraesCS3B01G285100) and four *dehydrin* genes (TraesCS6A01G350500, TraesCS6D01G332900, TraesCS5A01G369800, and TraesCS6D01G333600) in the roots and four *LEA* genes (TraesCS2B01G313900, TraesCS2D01G295700, TraesCS7D01G026300, and TraesCS2B01G232700) and two *dehydrin* genes (TraesCS5B01G426800 and TraesCS6B01G383800) in leaves of plants under salt stress (Additional file 2: Table S2 and Fig. 5). The KEGG enrichment analysis showed that only two photosynthesis pathway genes were differentially expressed under control and stress conditions (Additional file 1: Figure S4). Figure S4 showed that the two ferredoxin-NADP^+^ reductase (*petH*) genes were downregulated in leaves under LSS. The *petH* gene is critical photosynthesis gene that generates NADPH.

**Table 1:** Gene expression data involved in abscisic acid, gibberellin and jasmonic acid

		Leaf		Root	
Gene ID	Gene Name	Fold change	Adjusted *P* value	Fold change	Adjusted *P* value
TraesCS6A01G333600	*ABF*	4.5	0.000	2.1	0.000
TraesCS3A01G378700	*ABF*	10.3	0.000	2.7	0.000
TraesCS3B01G411300	*ABF*	6.1	0.000	3.1	0.000
TraesCS3D01G371900	*ABF*	50.5	0.000	2.6	0.001
TraesCS6B01G364000	*ABF*	6.1	0.000	1.8	0.000
TraesCS6D01G312800	*ABF*	4.3	0.000	1.6	0.000
TraesCS7A01G170600	*ABF*	7.4	0.000	0.8	0.473
TraesCS7B01G075600	*ABF*	13.6	0.000	0.8	0.485
TraesCS7D01G171300	*ABF*	7.8	0.000	0.6	0.019
TraesCS3A01G233000	*DELLA*	18.3	0.014	0.7	0.051
TraesCS3D01G220100	*DELLA*	49.5	0.004	0.7	0.089
TraesCS3D01G385500	*DELLA*			5.2	0.001
TraesCS4A01G007800	*JAZ*	8.3	0.000	0.1	0.000
TraesCS4D01G296000	*JAZ*	5.1	0.000	0.1	0.000
TraesCS7A01G201100	*JAZ*	6.9	0.000	0	0.000
TraesCS7A01G201200	*JAZ*	11.9	0.002	0	0.000
TraesCS7A01G201400	*JAZ*	8.8	0.001	0	0.000
TraesCS7A01G201500	*JAZ*	6.9	0.013	0	0.000
TraesCS7B01G107700	*JAZ*	6.8	0.000	0	0.000
TraesCS7B01G107800	*JAZ*	6.7	0.003	0	0.000
TraesCS7B01G107900	*JAZ*	8.7	0.000	0	0.000
TraesCS7B01G108000	*JAZ*	4.6	0.015	0	0.000
TraesCS2D01G285300	*JAZ*	4.9	0.022	0.1	0.000
TraesCS4A01G007900	*JAZ*	1.6	0.621	0.2	0.000
TraesCS4B01G297000	*JAZ*	1.4	0.674	0.3	0.000
TraesCS4B01G297100	*JAZ*	2.3	0.116	0.2	0.000
TraesCS4D01G295900	*JAZ*	0.8	0.730	0.3	0.000
TraesCS5A01G204900	*JAZ*	0.7	0.337	0.4	0.000
TraesCS5B01G211000	*JAZ*	1	0.977	0.1	0.000
TraesCS7A01G201600	*JAZ*	7.4	0.081	0	0.000
TraesCS7B01G108200	*JAZ*	2.7	0.084	0.1	0.001
TraesCS7B01G108300	*JAZ*	3.5	0.147	0	0.000
TraesCS7B01G108400	*JAZ*	10.1	0.001	0	0.000
TraesCS7B01G108500	*JAZ*	13.9	0.010	0	0.000
TraesCS7D01G204400	*JAZ*	6.7	0.045	0	0.000
TraesCS7D01G204500	*JAZ*	3.1	0.243	0	0.000
TraesCS7D01G204600	*JAZ*	4.9	0.166	0	0.000
TraesCS7D01G204700	*JAZ*	6.2	0.033	0	0.000

Fold change = stress/control. ABF, ABRE-binding factor; JAZ, Jasmonate ZIM-domain. The wheat seeds were treated with 100 mM NaCl for 30 days. Each treatment had three biological replicates

### Effects of long-term salinity stress on the relative expression of homeologs

**Fig. 6:**
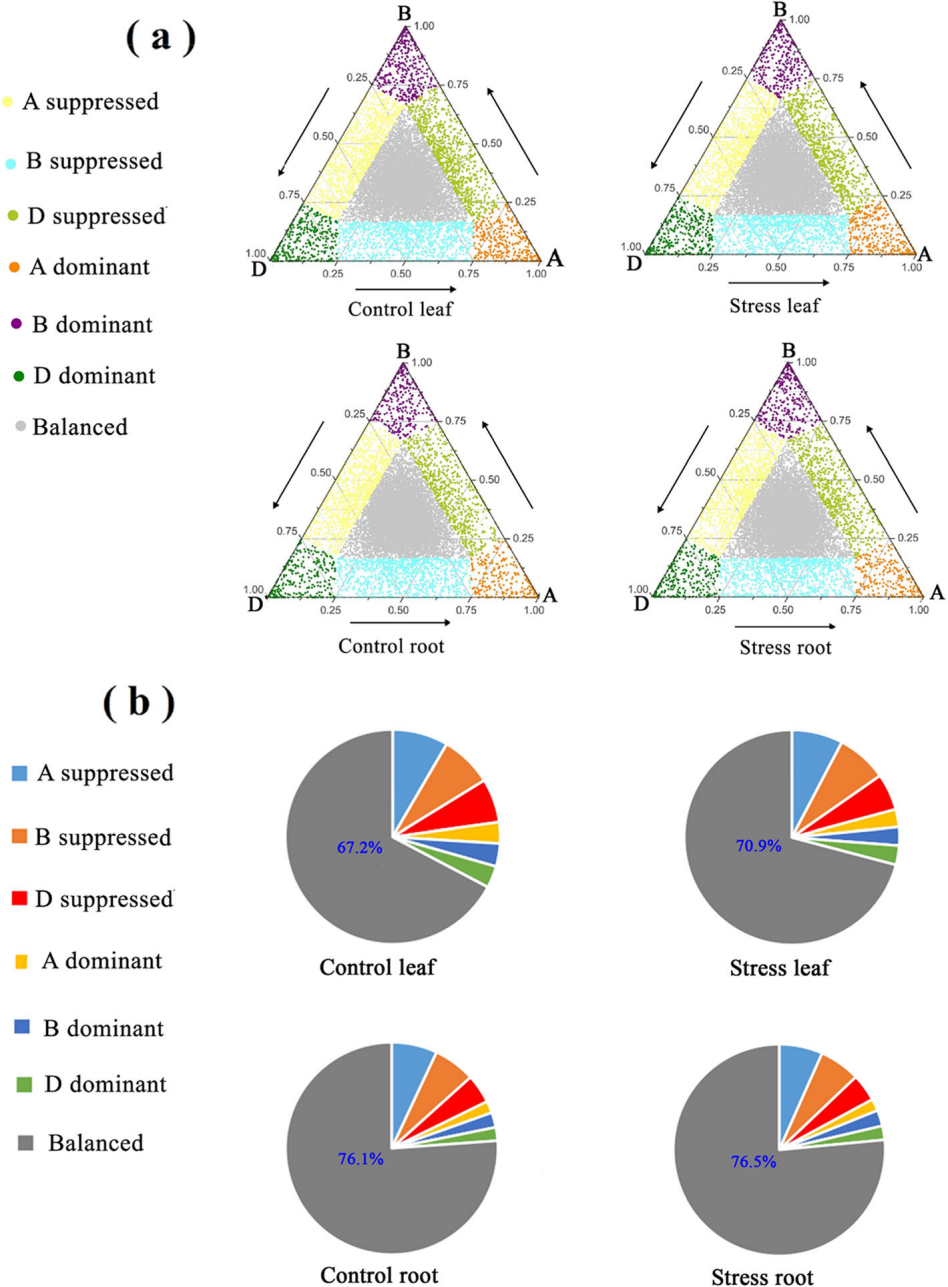
Effects of long-term salinity stress on homeolog expression bias in all triads. **a** Ternary plot displaying homeologous expression bias categories (HEBCs) of all triads. **b** Percentage of each HEBC on total triads. The wheat seeds were treated with 100 mM NaCl for 30 days. The three vertexes (A, B and D) of the triangle represent ideally dominant expression of the corresponding A, B and D homeologs, respectively

**Fig. 7:**
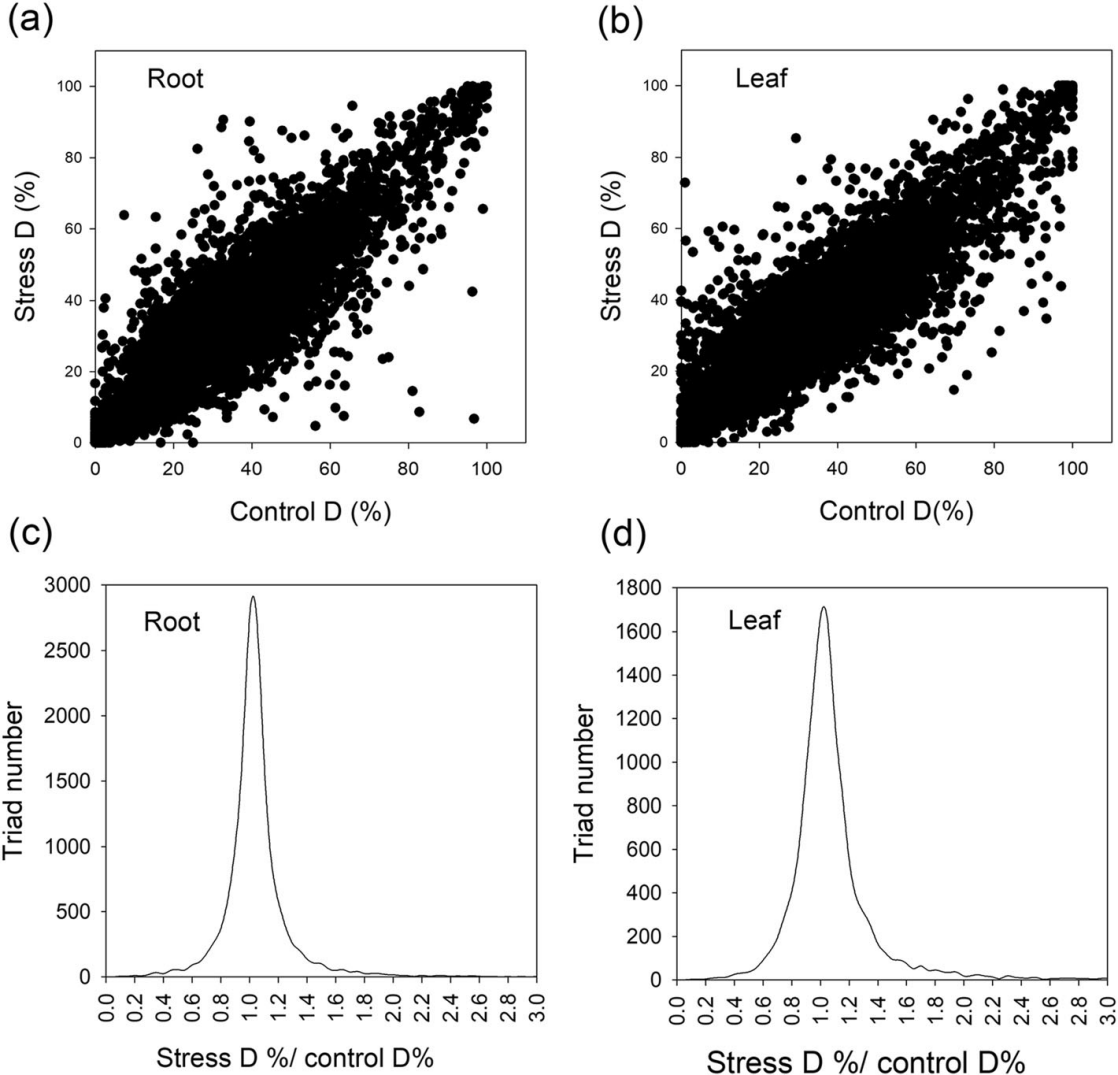
Effects of long-term salinity stress on relative expression of D homeolog (D%) for all triads. **a** Rectangular coordinate plot showing relationship between control D% (x axis) and stress D% (y axis) for all the triads. Transcripts per million reads (TPM) values was used to represent the absolute gene expression level of each gene. The relative expression value of the D homeolog (D%) of a gene triad was calculated by the following formula: TPM_D-homeolog_/(TPM_A-homeolog_ + TPM_B-homeolog_ + TPM_D-homeolog_). **b** Distribution plot showing ratios of stress D%/control D%. The wheat seeds were treated with 100 mM NaCl for 30 days

We detected the expression of 16,438 triads (Fig. 6). We calculated the relative expression value (REV) of three homeologs within a given triad for all detected triads. For example, we calculated D homeolog REV (D%) of a given triad using the following equation: D% = TPM_D_*100%/(TPM_A_ + TPM_B_ + TPM_D_), where TPM is the absolute TPM value of each homeolog within a given triad. Here, we were particularly interested in changes in D% between control and stress conditions (Fig. 7). To test how LSS affected D%, we compared the difference in D% between control and LSS conditions (Fig. 7a-b), and plotted control D% (x axis) and stress D% (y axis) for all the triads (Fig. 7a-b). We found that the majority of the data points were associated with the diagonal line, indicating a small effect of LSS on the relative expression of the D homeolog. We also calculated the ratios of control D% and LSS D% for all triads and obtained a probability distribution map for the ratios (Fig. 7c-d). With a ≥ 20% fold change (ratio of control D% and LSS D%) being defined as a significant effect of LSS on the relative expression of the homeolog, the D% difference between control and LSS plants was < 20% in 77% of root triads and 70% of leaf triads (Fig. 7c-d). Similarly, in only 23–32% of triads did LSS conditions result in a significant effect on the relative expression of the A or B homeolog (Additional file 2: Table S3). Taken together, LSS caused small effects on the relative expression of homeologs for most triads.

**Fig. 8:**
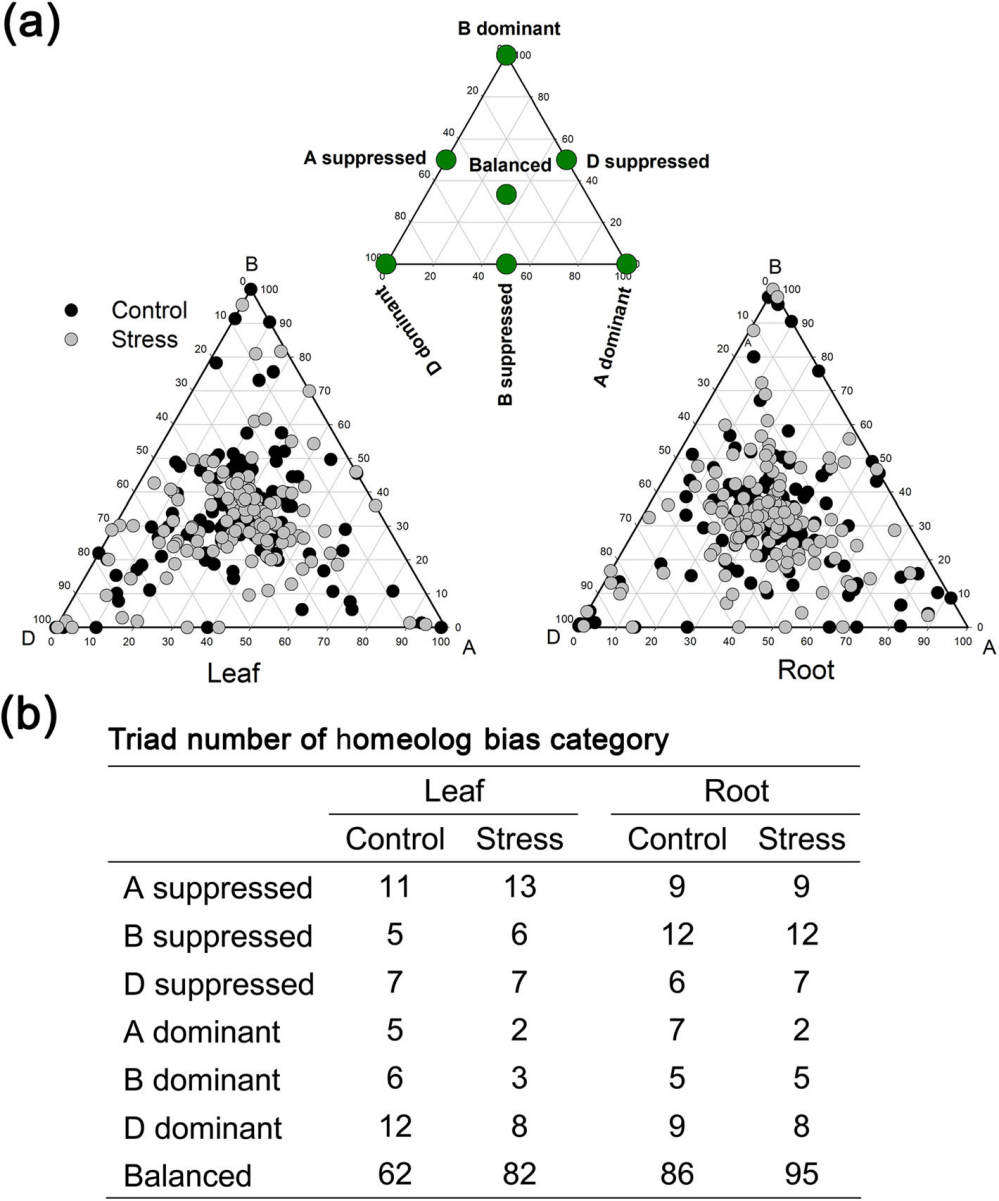
Effects of long-term salinity stress on homeolog expression bias in the salinity-tolerant triads. **a** Ternary plot displaying homeologous expression bias categories (HEBCs) of all the salinity-tolerant triads. **b** Triad number of each HEBC for all the salinity-tolerant triads

On the basis of REVs of homeologs of each triad, we used the methods of Ramírez-González et al. (2018) to assign all triads into seven homeolog expression bias categories (Figs. 6a and 8a) [14]. The three vertices (A, B and D) of the triangle represent the ideally dominant expression of the corresponding homeologs. For example, in the triad represented by vertex A of the triangle, B homeolog and D homeolog make 0% contribution to the total expression of the three homeologs of this triad, and A homeolog makes 100% contribution. The circle close to the vertices showed the dominant homeolog expression. Figure 6b showed that “balanced” is the dominant homeolog expression bias category (HEBC) with a percentage of 67–77%. The percentage of the “suppressed” category (4.2–8.3%) is significantly greater than that of the “dominant” category (2.0–3.5%). The percentage of “D suppressed” is less than that of “B suppressed” and “A suppressed” under both conditions. Finally, we considered whether LSS changed the HEBC for all the triads detected (Additional file 2: Table S4). In leaves, 17.4% of the triads showed stress-induced changes in HEBC, whereas, in the roots, only 11.0% of the triads showed stress-induced changes in HEBC (Additional file 2: Table S4).

### Expression response of homeologs to long-term salinity stress in salinity-tolerant triads

Initially, we considered the changes in absolute expression of homeologs under LSS for salinity-tolerant gene triads. We determined the fold change (stress/control) values of absolute expression of homeologs from the transcriptomic profiling generated by the DESeq2 R package. Rectangular coordinate plots were drawn with x = A homeolog fold change and y = D homeolog fold change for all salt-tolerant triads, as well as for x = B homeolog fold change and y = D homeolog fold change, and x = A homeolog fold change and y = B homeolog fold change (Additional file 1: Figure S5). For most salinity-tolerant triads, the fold change values (stress/control) of A, B and D homeologs were similar. However, in several salinity-tolerant triads, different homeologs within a triad showed different fold change values (Additional file 2: Table S5 and Additional file 1: Figure S5–6). In some triads, such as *LEA* (Triad1212) and *LEA* (Triad5607), fold changes of the D-homeolog were much greater than those of the A- and B-homeologs (Additional file 1: Figure S6 and Additional file 2: Table S5). In some salinity-tolerant triads, such as *LEA* (Triad14049), *LEA* (Triad14050), *LEA* (Triad10483), *LEA* (Triad14474), and *potassium transporter* gene (Triad16538), the fold changes of the B-homeolog were much greater than those of the A-homeolog and D-homeolog (Additional file 1: Figure S6). In *LEA* (Triad14048), *LEA* (Triad14050), *LEA* (Triad10483), *LEA* (Triad14474), and *potassium transporter* gene (Triad16538), the A-homeolog showed fold changes markedly greater than those of the B-homeolog and D-homeolog (Additional file 1: Figure S6).

Next, we focused on the effects of LSS on the HEBC of the salinity-tolerant triads based on REVs of the homeologs. We found that, under LSS conditions, the salinity-tolerant triad numbers of D-dominant HEBCs was much greater than those of A-dominant and B-dominant HEBCs. In LSS-stressed roots, eight salinity-tolerant gene triads showed D-dominance (Fig. 8b), namely three *LEA* genes, one *dehydrin* gene, two *potassium transporter* genes and two *aquaporin* genes (Additional file 2: Table S5). In LSS-stressed leaves, eight salinity-tolerant gene triads showed D-dominance (Fig. 8b), namely the five *LEA* genes, one *dehydrin* gene, two sodium/cation exchanger gene and one aquaporin gene (Additional file 2: Table S5). We also compared the HEBC of salinity-tolerant triads under control and stress conditions and found that the HEBC values of few salinity-tolerant triads were altered by LSS (Additional file 2: Table S5). The changes in HEBCs of several *LEA* gene triads in leaf in ternary plots were revealed (Fig. 5b). The results showed that LSS conditions shifted the HEBCs of Triad4081 and Triad1565 from D-D to balanced, and that of Triad901 from D-D to B-S, and that of Triad4901 from D-D to B-S, whereas the HEBCs of Triad105 remained unchanged.

### qRT-PCR validation

The results of RNA-Seq (RNA sequencing) were validated by qRT-PCR (Additional file 2: Table S6). The results showed that 8 out of 10 selected genes showed consistent results between qRT-PCR and RNA-Seq (Additional file 2: Table S6), confirming that the results from RNA-Seq were reliable.

## Discussion

### Osmotic adjustment and tissue tolerance

Plants employ various traits and mechanisms to protect themselves against the damaging effects of salts in saline soil. The principal mechanisms are osmotic adjustment, detoxification of reactive oxygen species (ROS) and tissue tolerance. Because the accumulation of organic solutes has a much higher energy cost than that of Na^+^ or Cl^−^, under salinity stress, tolerant crop varieties or halophytic species usually accumulate the inorganic ions as osmotic regulators [15, 16]. In order to alleviate the toxicity of these ions, these salinity-tolerant plants will compartmentalize Na^+^ or Cl^−^ in the vacuole to reduce their concentrations in the cytoplasm to below toxic levels, and will also accumulate compatible solutes and K^+^ in the cytoplasm to balance the osmotic pressure caused by Na^+^ or Cl^−^ in the vacuole. For cells of a mature leaf or root, given that the cytoplasm represents a very small proportion of the cell volume, a relatively small absolute amount of organic solute in the cytoplasm can produce the high molarity required to mitigate the osmotic stress from the vacuole. Our results showed that, under LSS, the concentrations of amino acids were particularly enhanced in the leaves, but not in the roots. In contrast, under LSS, increased carbohydrate concentration was more apparent in the roots than in the leaves (Additional file 2: Table S1). Alanine, proline, maltose and sucrose were the dominant compatible solutes in allohexaploid wheat leaves, whereas alanine, fructose, glucose and sucrose were the dominant compatible solutes in allohexaploid wheat roots. Taken together, amino acids and carbohydrates made similar contributions to increasing the osmotic potential of the leaf cytoplasm, whereas carbohydrates played more important roles in osmotic adjustment of the root cytoplasm than did amino acids.

A common tissue tolerance mechanism is the Na^+^-exclusion strategy mediated by the *HKT1* gene in both crops and *Arabidopsis* [13, 17, 18]. The *TaHKT1;5* gene was identified to modulate Na^+^ exclusion in allohexaploid wheat [10, 17]. In the present work, we found that the D homeolog of the *TaHKT1;5* gene (TraesCS4D01G361300) was greatly up-regulated in roots under LSS (Additional file 2: Table S2), a result which was consistent with the finding of Yang et al. 2014 [8]. Additionally, we observed marked up-regulation of many other critical salinity-tolerance genes in roots under LSS, such as four *NHX* genes, four *HKT* genes, two *V-H*^*+*^*ATPase* genes, one *potassium channel SKOR* gene and three *potassium transporter* genes (Additional file 2: Table S2). One *SOS1* gene was also up-regulated in the roots (Additional file 2: Table S2). We suggest that these salinity tolerance genes may play essential roles in tissue tolerance and ion homeostasis in wheat roots under LSS. Interestingly, we observed that two aquaporin *TIP2;3* genes were markedly up-regulated in leaves but not in roots (Additional file 2: Table S2). In *Arabidopsis*, *PIP2;1* and *PIP2;2* aquaporins function as nonselective cation channels (NSCCs) that transport all cations into cells [19]. In stressed wheat leaves, the aquaporin *TIP2;3* genes many facilitate K^+^ accumulation in cells to improve the salinity tolerance of wheat.

In terms of osmotic adjustment, we observed that 69 *late embryogenesis abundant* (*LEA*) genes and 39 *dehydrin* genes were significantly up-regulated in either roots or leaves. The LEA proteins and dehydrin proteins are proposed to play critical roles in osmotic adjustment [20–23]. The number of *LEA* and *dehydrin* genes in wheat is much greater than in other plants. Wheat has at least 57 *dehydrin* genes and 429 *LEA* genes, while other plant species have about 15 *dehydrin* genes and 100 *LEA* genes [22]. Co-expression of a very high number of *dehydrin* and *LEA* genes should exhibit dosage effects in LSS response. Another interesting response to osmotic stress is related to aerenchyma and wax synthesis. Under LSS, the wheat leaf had less aerenchyma than under control conditions, which would reduce the frequency of water loss, but simultaneously limit the gas exchange rate of photosynthesis. Under salinity stress or drought stress, wax is synthesized and applied to the leaf cuticle to reduce water loss [24–27]. In the present work, up-regulated DEGs in the leaf were significantly enriched with respect to wax biosynthesis (Additional file 1: Figure S1 and S3). In addition, flavonoid biosynthesis genes were greatly up-regulated in the leaf under LSS (Additional file 1: Figure S1), and flavonoids would probably play roles in the detoxification of the ROS generated during the response of wheat to LSS.

### Energy partitioning mediated by plant hormones may be important for wheat salinity tolerance

The energy generated by photosynthesis is distributed among three major biological processes: biomass accumulation, general maintenance of metabolic processes, and response to environmental stress [15, 28, 29]. The majority of the energy is used by plants in general maintenance, with only a small proportion (10–40%) being partitioned for biomass accumulation [15]. Increasing soil salinity significantly promotes the shift of the energy from biomass accumulation to stress response [15, 28, 29]. For example, one strategy for restricting metabolism and growth is stomatal closure, mediated by the ABA signaling pathway, which can decrease the transpiration flow and concomitant Na^+^ influx into the leaf. It is well known that a plant retains only 1–5% of the water taken up by the root, with 95–99% of the water being used for transpiration. Under salinity stress, transpiration flow will carry large amounts of Na^+^ and Cl^−^, but most of these toxic ions need to be excluded into the rhizosphere solution, which will incur a marked energy cost [15, 16].

ABA signaling mediates a decrease in stomatal conductance, which reduces the influx of water and toxic ions into above-ground parts of plants, and, as a result, reduces the energy expenditure required to exclude Na^+^ and Cl^−^ [15, 16]. The results from the current study showed that LSS induced ABA accumulation in the leaves of allohexaploid wheat (Fig. 4), and DEGs were significantly enriched with respect to plant hormone signaling, particularly involving ABA, GA and JA (Additional file 1: Figure S1-S2). We observed that nine ABF (ABA response element binding factor) transcription factor genes, representing the final function of the ABA signaling system, were greatly up-regulated in wheat leaves under LSS (Table 1). Up-regulation of ABF genes can not only mediate stomatal closure and limit growth but also up-regulate the expression of the salinity-responsive genes that contain ABA-responsive elements (ABRE) in their promoter regions [30, 31]. LSS also induced a reduction in JA and GA_3_ concentrations and the up-regulation of three *DELLA* genes and 10 *JAZ* genes in wheat leaves (Table 1). DELLA and JAZ suppress the pathways mediated by gibberellins and jasmonic acid, respectively. According to these hormone expression data, we propose that, under LSS, wheat might shift energy from general maintenance to stress response through enhancing ABA pathways and suppressing the pathways controlled by GAs and JA in the leaf. Here, we proposed the hypothesis that GA and JA negatively modulate the LSS response of allohexaploid wheat, which could be tested by the exogenous application of GAs and/or JA and the analysis of wheat lines exhibiting overexpression or knockout of wheat *DELLA* and *JAZ* genes. Moreover, we observed that the thylakoids in chloroplasts of stressed wheat plants were at a higher density than those in the control wheat plants. We also observed downregulation of the two *petH* genes that generate NADPH during photosynthetic light reaction (Figure S4). The proteins and pigments that function in the photochemical reactions of photosynthesis are embedded in the thylakoid membrane. The end products of these photochemical reactions occurring on the thylakoids are ATP and NADPH, which are used for carbon fixation reactions as well as biosynthesis of some salinity-response solutes such as fatty acids (substrate for wax synthesis), amino acids and betaine (compatible solutes). Higher-density thylakoids of salinity-stressed wheat plants may remedy the lower expression of the *petH* genes and benefit the generation of ATP and NADPH to fuel salinity stress responses.

### Homeolog expression bias under long-term salinity stress

Duplicate genes generated following polyploidy may have three evolutionary fates: functional diversification (subfunctionalization or neofunctionalization), gene silencing or loss, or retention of original or similar functions (dosage-sharing model) [32]. Functional diversification of duplicate genes may be an important factor in the success of polyploid species during their evolutionary history [32]. However, our results and those of Ramírez-González et al. (2018) and Xiao et al. (2020) all showed that most of triads displayed balanced HEBC (with the three homeologs having similar expression levels) under different stress conditions and in different tissues [14, 23]. Most of the duplicate genes or homeologous genes generated by hexaploidization may have retained their original (parental) functions in extant natural allohexaploid wheat. For those triads with balanced HEBC, the expression sum of the three homeologs may be additive, or similar to the expression level of either of its diploid or tetraploid ancestors, or even transgressive. The transgressive expression of triads may have contributed to the development of the high stress tolerance of allohexaploid wheat because many adaptive mechanisms of plants to stress conditions rely on the dosage effects. For example, plant salinity tolerance is related to the absolute abundance of some crucial salinity tolerance proteins such as LEA, DHN, HKT, AKT, SOS, H^+^-ATPase and NHX [11–13, 22].

Although most triads showed balanced HEBCs, we still expected that differences in the expression and function partitioning of homeologs across different stress conditions would be present in some triads. Unfortunately, function partitioning of homeologs is difficult to identify. Nevertheless, RNA-Seq experiments can readily dissect the expression partitioning of homeologs. In the present study, we focused on the response of homeologs to LSS. In the current study, only about 30% of gene triads showed stress-induced changes in relative expression of homeologs (Additional file 2: Table S3), which is not consistent with the findings of Dong and Adams (2011) [33], where more than 70% of the triads showed stress-induced changes in the relative expression levels of the duplicate genes under stress conditions. For those wheat gene triads without stress-induced changes in the relative expression of the homeologs, expression of the three homeologs may be co-regulated by shared *cis-*acting elements and *trans* factors during response to LSS.

Similar to general triads, most salinity-tolerant triads also showed balanced HEBCs (Fig. 8b) under both control and LSS conditions. Co-expression of the three homeologs of these salinity-tolerant triads may exhibit dosage effects in salinity stress response [23]. Interestingly, some salinity-tolerant triads showed stress-induced changes in HEBCs, even in many of the crucial salinity tolerance genes such as the *dehydrin*, *LEA*, *sodium/cation exchanger*, and *aquaporin* genes. The HEBC changes of salinity-tolerant triads caused by LSS was more frequent in leaves (28.57%) than in roots (13.8%). Surprisingly, for general triads, the percentage in leaves was only 17.7% (Additional file 2: Table S4). This implied that response mechanisms of the salinity-tolerant triads may be different from those of general triads in the leaves.

## Conclusions

The response of allohexaploid wheat to LSS has been shown to be a comprehensive and coordinated process occurring at different levels, including the molecular, organellar, cellular, tissue and whole-plant levels. Energy partitioning between the maintenance of general metabolism and stress response may be crucial for the survival of allohexaploid wheat under LSS. Under LSS, wheat appears to shift energy from general maintenance to stress response through enhancing ABA pathways and suppressing GA and JA pathways in leaves. Moreover, the increased thylakoid density in chloroplasts of stressed leaves will generate more ATP and NADPH to fuel the biosynthesis of salinity-responsive solutes. In addition, LSS influences homeolog expression bias of salinity-tolerant triads.

## Methods

### Plant growth and long-term salinity stress treatment

This experiment was conducted in the experimental garden of Northeast Normal University, Changchun, China. Thirty seeds of allohexaploid wheat (*Triticum aestivum* L., genome BBAADD, cv. Chinese Spring) were sown in each plastic pot containing thoroughly washed sand. The seeds of Chinese Spring wheat used in this work were kindly provided by Dr. Bao Liu (Northeast Normal University, China). Chinese Spring seeds were sown in 10 pots containing half-strength Hoagland nutrient solution as control group, and in parallel Chinese Spring seeds also were sown in another 10 pots containing stress treatment solution as stress treatment group. The half-strength Hoagland nutrient solution supplemented with 100 mM NaCl was used as salinity stress treatment solution. Control pots were watered with half-strength Hoagland nutrient solution, and stress treatment pots were watered with stress treatment solution. The stress treatment was applied from the seed germination stage to young seedling stage for 30 days. The experiment was conducted from mid-April to early June under a day/night temperature range of 18–25 °C/15–20 °C and a 14–15 h day photoperiod. All 20 pots were placed outdoors with protection from the rain. The experimental design was randomized complete block design.

### Leaf anatomy, chloroplast ultrastructure, and biochemical measurements

Mature leaves at the same leaf position were collected for chloroplast ultrastructure and leaf anatomy experiments. The chloroplast ultrastructure and leaf anatomy experiments were performed by a commercial company (Servicebio) using the workflow of Xiao et al. (2020) [23]. We collected the mature (functional) leaves at middle leaf position and root samples for biochemical measurements. Ten plants were pooled to make a biological replicate, and each treatment × tissue combination consisted of three biological replicates. All collected samples were freeze-dried for biochemical analyses. Concentrations of free amino acids and carbohydrates in freeze-dried samples were measured by a LC-MS-MS system (API3200MD, AB SCIEX) using the workflow of Zhao et al. (2017) [34]. Plant hormone concentrations of fresh samples were also measured by this LC-MS-MS system using the workflow of Xiao et al. (2020) [23]. Dried samples were digested three times in 65% HNO_3_ at 120 °C, and their Na^+^ and K^+^ contents were measured by an atomic absorption spectrophotometer (TAS-990super, PERSEE, China).

### RNA sequencing and qRT-PCR

We collected the mature (functional) leaves at middle leaf position and root samples from 30-d-old wheat seedlings for qRT-PCR and RNA sequencing experiments. Mature leaves at the same leaf position for each treatment were chosen for the further experiments. Ten plants were pooled to represent a biological replicate, and each treatment had three biological replicates. We performed RNA sequencing and qRT-PCR experiments using method of Xiao et al. (2020) [23]. We downloaded wheat reference genome (iwgsc_refseqv1.0) from the International Wheat Genome Sequencing Consortium homepage (http://www.wheatgenome.org). We defined DEGs as having an adjusted *P* value ≤0.05 and |log2fold change| ≥ 1. The DEGs were exposed to KEGG enrichment by using the hypergeometric test with adjusted *P* values (Fisher test). Ten salinity tolerance genes were randomly chosen in qRT-PCR experiment to validate the results of RNA sequencing. The sequences of the gene-specific primers were showed in Table S6. *Actin* and *RLI* were used as internal control genes [35, 36]. The relative gene expression level was calculated by the △△Ct method [37].

Based on the wheat reference genome (iwgsc_refseqv1.0), 17,400 syntenic and 1074 nonsyntenic triads were identified by Ramírez-González et al. (2018) [14]. Each gene triad is composed of one A homeolog, one B homeolog, and one D homeolog. We used TPM values to show the absolute expression value of each gene [14]. We also used the method of Ramírez-González et al. (2018) to define and calculate homeolog expression bias categories for each triad [14].

### Statistical analysis and experimental design

The experimental design was a randomized complete block design, with each treatment having three biological replicates. The statistical significance of phytochemical measurements and qRT-PCR were determined by the *t-*test at the α0.05 level, using SPSS version 16.0 (IBM). Statistical test of RNA-Seq data was performed by DESeq2 R package (1.20.0).

## Supplementary information

**Additional file 1: Figure S1**. Kyoto Encyclopedia of Genes and Genomes (KEGG) enrichment of differentially expressed genes in allohexaploid wheat leaf. Top 20 KEGG pathways with adjusted *P* value<0.05 are displayed. **Figure S2**. Kyoto Encyclopedia of Genes and Genomes (KEGG) enrichment of differentially expressed genes in allohexaploid wheat root. KEGG pathways with adjusted *P* value<0.05 are displayed. **Figure S3**. Effects of long-term salinity stress on gene expression involved in wax biosynthesis. (a) Gene expression change was marked on the pathway of wax biosynthesis, and the red box indicates up-regulated expression under long-term salinity stress. The wax biosynthesis pathway diagram was adapted from the diagram of KEGG website. (b) Gene expression data involved in wax metabolism in wheat leaf under long-term salinity stress. FAR, alcohol-forming fatty acyl-CoA reductase. MAH1, midchain alkane hydroxylase; CYP96A15; WSD1, wax-ester synthase/diacylglycerol *O*-acyltransferase. Fold change = stress/control, Q value is the adjusted *P* value using the Benjamini-Hochberg method. **Figure S4.** Effects of long-term salinity stress on expression of two ferredoxin-NADP^+^ reductase (*petH*) genes in wheat leaves. **Figure S5**. Relationships among A, B and D homeologs in terms of expression fold change (stress/control) for all salinity-tolerant triads. **Figure S6.** Expression fold change (stress/control) of A, B and D homeologs of 15 typical salinity-tolerant triads. * indicates significant difference (adjusted *P* value < 0.05 and |log2fold change| ≥ 1).

**Additional file 2: Table S1**. Fold change and percent contribution to total molarity of each compatible solute. Fold change is ratio of stress /control. Percent contribution is calculated with following equation: Percent contribution of a given solute = its molarity content (μmol g-1 DW) × 100/total molarity content, where total molarity content is sum of molarity contents of all 37 solutes. The wheat seeds were treated with 100 mM NaCl for 30 days. CL, control leaf; SL, stress leaf; CR, control root; SR, stress root. **Table S2**. Expression data of salinity-tolerant genes involved in osmotic adjustment, ion homeostasis and tissue tolerance. **Table S3**. Percentage of the triad showing stress-induced significant change in relative expression of homeolog. Significant change was defined as ≥20% difference between stress and control in relative expression of homeologs. **Table S4.** Number of triads showing differential homeolog expression bias categories between control and stress treatments. **Table S5.** Expression data and homeolog expression bias categories of salinity-tolerant triads involved in osmotic adjustment, ion homeostasis and tissue tolerance. A-S, A suppressed; A-D, A dominant; B-S, B suppressed; B-D, B dominant; D-S, D suppressed; D-D, D dominant. **Table S6**: Results of qRT-PCR of wheat leaves. Each treatment had three biological replicates.

## Data Availability

About 10 Gb of clean data from each sample were used to perform the RNA-Seq experiment. All raw data of transcriptional analysis are deposited at NCBI (Accession nos. SRR10177014, SRR10177013, SRR10177012, SRR10177011, SRR10177010, SRR10177009, SRR10177008, SRR10177007, SRR10177006, SRR10177005, SRR10177004, and SRR10177003). The datasets used and/or analyzed during the current study are available from the corresponding author on request. All SRA data used in this work are available at following links: SRR10177014: https://www.ncbi.nlm.nih.gov/sra/?term=SRR10177014 SRR10177013: https://www.ncbi.nlm.nih.gov/sra/?term=SRR10177013 SRR10177012: https://www.ncbi.nlm.nih.gov/sra/?term=SRR10177012 SRR10177011: https://www.ncbi.nlm.nih.gov/sra/?term=SRR10177011 SRR10177010: https://www.ncbi.nlm.nih.gov/sra/?term=SRR10177010 SRR10177009: https://www.ncbi.nlm.nih.gov/sra/?term=SRR10177009 SRR10177008: https://www.ncbi.nlm.nih.gov/sra/?term=SRR10177008 SRR10177007: https://www.ncbi.nlm.nih.gov/sra/?term=SRR10177007 SRR10177006: https://www.ncbi.nlm.nih.gov/sra/?term=SRR10177006 SRR10177005: https://www.ncbi.nlm.nih.gov/sra/?term=SRR10177005 SRR10177004: https://www.ncbi.nlm.nih.gov/sra/?term=SRR10177004 SRR10177003: https://www.ncbi.nlm.nih.gov/sra/?term=SRR10177003

## Abbreviations

LSS: Long-term salinity stress
HEBC: Homeolog expression bias category
SA: Salicylic acid
GA: Gibberellin
IAA: Indole-3-acetic acid
ABA: Abscisic acid
DEG: Differentially expressed gene
REV: Relative expression value
D%: D homeolog relative expression value
S: Suppressed
D: Dominant
HEBC: Homeolog expression bias category
ABF: ABA response element binding factor
JAZ: Jasmonate ZIM-domain
LSS: Long-term salinity stress
LEA: Late embryogenesis abundant
DHN: Dehydrin
K_trans: Potassium transporter
V-H_PPase: Vacuolar-H^+^-pyrophosphatase
NCX: Sodium/calcium exchanger
KEA: K efflux antiporter
