# Supplementary figures and images for: Adaptive strategy of allohexaploid wheat to long-term salinity stress
Source: BMC Plant Biol. 2020 May 12;20:210. doi: 10.1186/s12870-020-02423-2 (PMC7216640; doi:10.1186/s12870-020-02423-2)

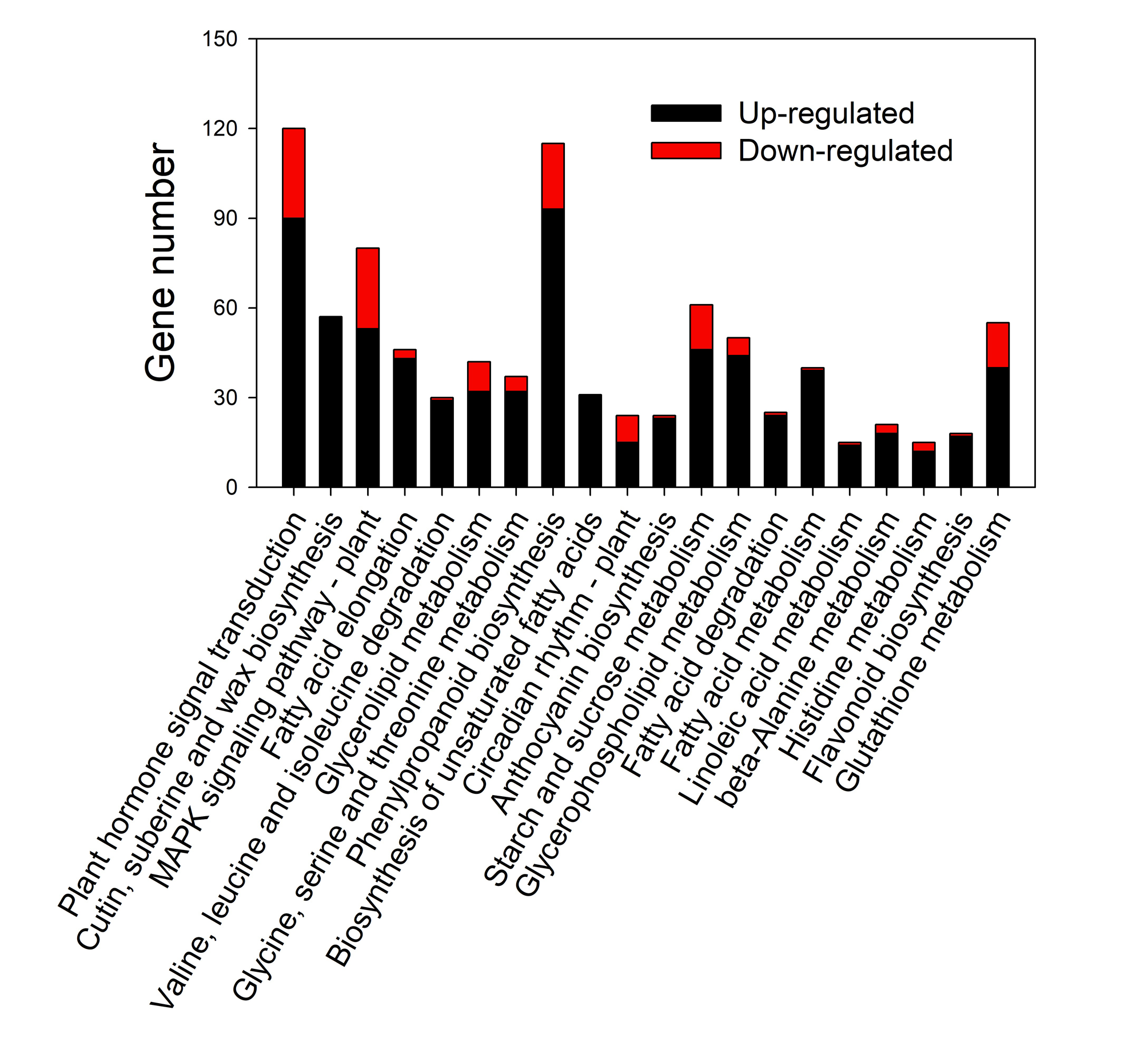

Supplement: Supplementary file 1 — Additional file 1: Figure S1. Kyoto Encyclopedia of Genes and Genomes (KEGG) enrichment of differentially expressed genes in allohexaploid wheat leaf. Top 20 KEGG pathways with adjusted P value<0.05 are displayed. Figure S2. Kyoto Encyclopedia of Genes and Genomes (KEGG) enrichment of differentially expressed genes in allohexaploid wheat root. KEGG pathways with adjusted P value<0.05 are displayed. Figure S3. Effects of long-term salinity stress on gene expression involved in wax biosynthesis. (a) Gene expression change was marked on the pathway of wax biosynthesis, and the red box indicates up-regulated expression under long-term salinity stress. The wax biosynthesis pathway diagram was adapted from the diagram of KEGG website. (b) Gene expression data involved in wax metabolism in wheat leaf under long-term salinity stress. FAR, alcohol-forming fatty acyl-CoA reductase. MAH1, midchain alkane hydroxylase; CYP96A15; WSD1, wax-ester synthase/diacylglycerol O-acyltransferase. Fold change = stress/control, Q value is the adjusted P value using the Benjamini-Hochberg method. Figure S4. Effects of long-term salinity stress on expression of two ferredoxin-NADP+ reductase (petH) genes in wheat leaves. Figure S5. Relationships among A, B and D homeologs in terms of expression fold change (stress/control) for all salinity-tolerant triads. Figure S6. Expression fold change (stress/control) of A, B and D homeologs of 15 typical salinity-tolerant triads. * indicates significant difference (adjusted P value < 0.05 and |log2fold change| ≥ 1). [file 12870_2020_2423_MOESM1_ESM.zip › Figure S1 Leaf KEGG TPM0.tif]

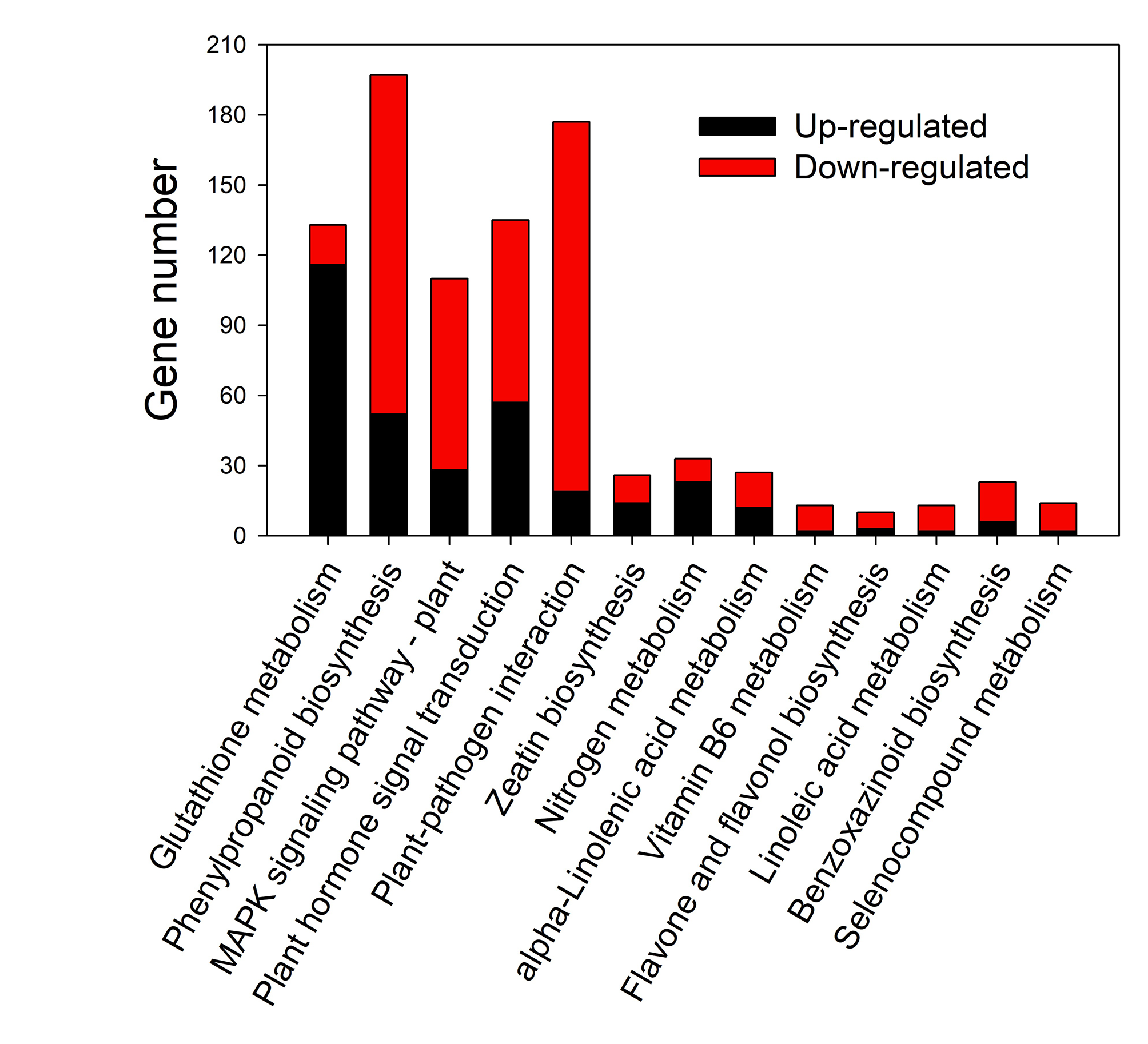

Supplement: Supplementary file 1 — Additional file 1: Figure S1. Kyoto Encyclopedia of Genes and Genomes (KEGG) enrichment of differentially expressed genes in allohexaploid wheat leaf. Top 20 KEGG pathways with adjusted P value<0.05 are displayed. Figure S2. Kyoto Encyclopedia of Genes and Genomes (KEGG) enrichment of differentially expressed genes in allohexaploid wheat root. KEGG pathways with adjusted P value<0.05 are displayed. Figure S3. Effects of long-term salinity stress on gene expression involved in wax biosynthesis. (a) Gene expression change was marked on the pathway of wax biosynthesis, and the red box indicates up-regulated expression under long-term salinity stress. The wax biosynthesis pathway diagram was adapted from the diagram of KEGG website. (b) Gene expression data involved in wax metabolism in wheat leaf under long-term salinity stress. FAR, alcohol-forming fatty acyl-CoA reductase. MAH1, midchain alkane hydroxylase; CYP96A15; WSD1, wax-ester synthase/diacylglycerol O-acyltransferase. Fold change = stress/control, Q value is the adjusted P value using the Benjamini-Hochberg method. Figure S4. Effects of long-term salinity stress on expression of two ferredoxin-NADP+ reductase (petH) genes in wheat leaves. Figure S5. Relationships among A, B and D homeologs in terms of expression fold change (stress/control) for all salinity-tolerant triads. Figure S6. Expression fold change (stress/control) of A, B and D homeologs of 15 typical salinity-tolerant triads. * indicates significant difference (adjusted P value < 0.05 and |log2fold change| ≥ 1). [file 12870_2020_2423_MOESM1_ESM.zip › Figure S2 ROOT KEGG.tif]

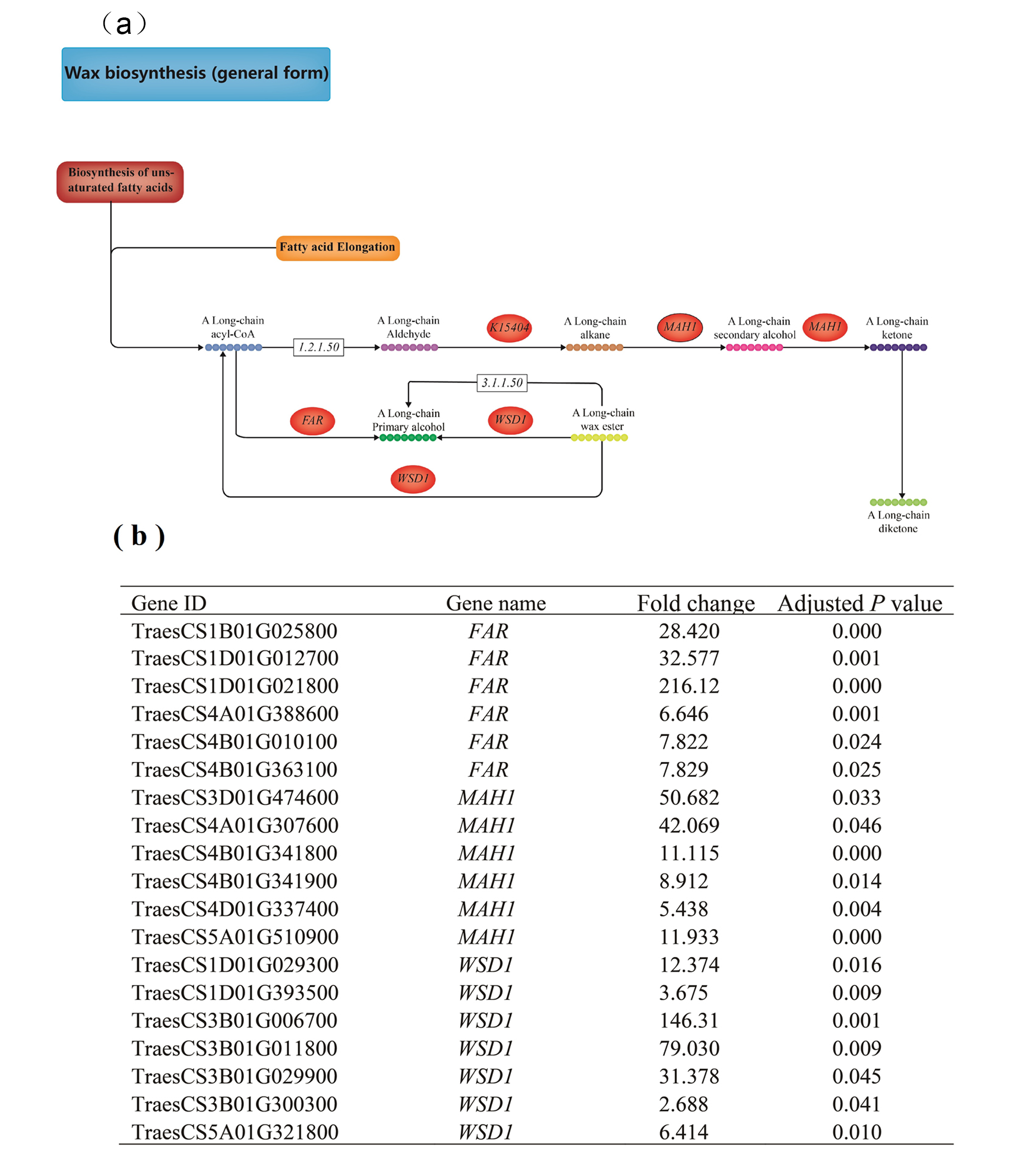

Supplement: Supplementary file 1 — Additional file 1: Figure S1. Kyoto Encyclopedia of Genes and Genomes (KEGG) enrichment of differentially expressed genes in allohexaploid wheat leaf. Top 20 KEGG pathways with adjusted P value<0.05 are displayed. Figure S2. Kyoto Encyclopedia of Genes and Genomes (KEGG) enrichment of differentially expressed genes in allohexaploid wheat root. KEGG pathways with adjusted P value<0.05 are displayed. Figure S3. Effects of long-term salinity stress on gene expression involved in wax biosynthesis. (a) Gene expression change was marked on the pathway of wax biosynthesis, and the red box indicates up-regulated expression under long-term salinity stress. The wax biosynthesis pathway diagram was adapted from the diagram of KEGG website. (b) Gene expression data involved in wax metabolism in wheat leaf under long-term salinity stress. FAR, alcohol-forming fatty acyl-CoA reductase. MAH1, midchain alkane hydroxylase; CYP96A15; WSD1, wax-ester synthase/diacylglycerol O-acyltransferase. Fold change = stress/control, Q value is the adjusted P value using the Benjamini-Hochberg method. Figure S4. Effects of long-term salinity stress on expression of two ferredoxin-NADP+ reductase (petH) genes in wheat leaves. Figure S5. Relationships among A, B and D homeologs in terms of expression fold change (stress/control) for all salinity-tolerant triads. Figure S6. Expression fold change (stress/control) of A, B and D homeologs of 15 typical salinity-tolerant triads. * indicates significant difference (adjusted P value < 0.05 and |log2fold change| ≥ 1). [file 12870_2020_2423_MOESM1_ESM.zip › Figure S3 wax pathway revised.tif]

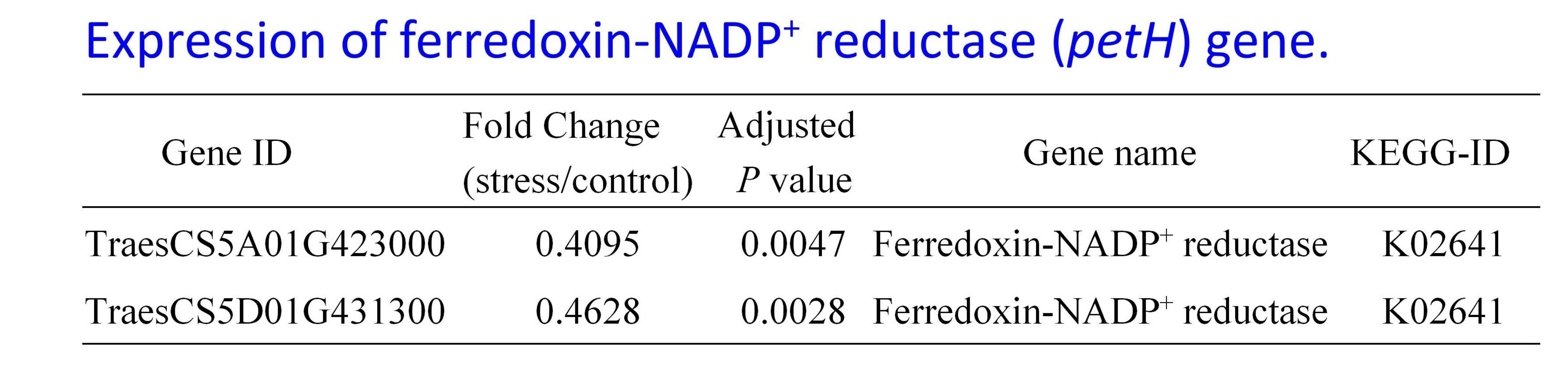

Supplement: Supplementary file 1 — Additional file 1: Figure S1. Kyoto Encyclopedia of Genes and Genomes (KEGG) enrichment of differentially expressed genes in allohexaploid wheat leaf. Top 20 KEGG pathways with adjusted P value<0.05 are displayed. Figure S2. Kyoto Encyclopedia of Genes and Genomes (KEGG) enrichment of differentially expressed genes in allohexaploid wheat root. KEGG pathways with adjusted P value<0.05 are displayed. Figure S3. Effects of long-term salinity stress on gene expression involved in wax biosynthesis. (a) Gene expression change was marked on the pathway of wax biosynthesis, and the red box indicates up-regulated expression under long-term salinity stress. The wax biosynthesis pathway diagram was adapted from the diagram of KEGG website. (b) Gene expression data involved in wax metabolism in wheat leaf under long-term salinity stress. FAR, alcohol-forming fatty acyl-CoA reductase. MAH1, midchain alkane hydroxylase; CYP96A15; WSD1, wax-ester synthase/diacylglycerol O-acyltransferase. Fold change = stress/control, Q value is the adjusted P value using the Benjamini-Hochberg method. Figure S4. Effects of long-term salinity stress on expression of two ferredoxin-NADP+ reductase (petH) genes in wheat leaves. Figure S5. Relationships among A, B and D homeologs in terms of expression fold change (stress/control) for all salinity-tolerant triads. Figure S6. Expression fold change (stress/control) of A, B and D homeologs of 15 typical salinity-tolerant triads. * indicates significant difference (adjusted P value < 0.05 and |log2fold change| ≥ 1). [file 12870_2020_2423_MOESM1_ESM.zip › Figure S4 photosynthesis revised.jpg]

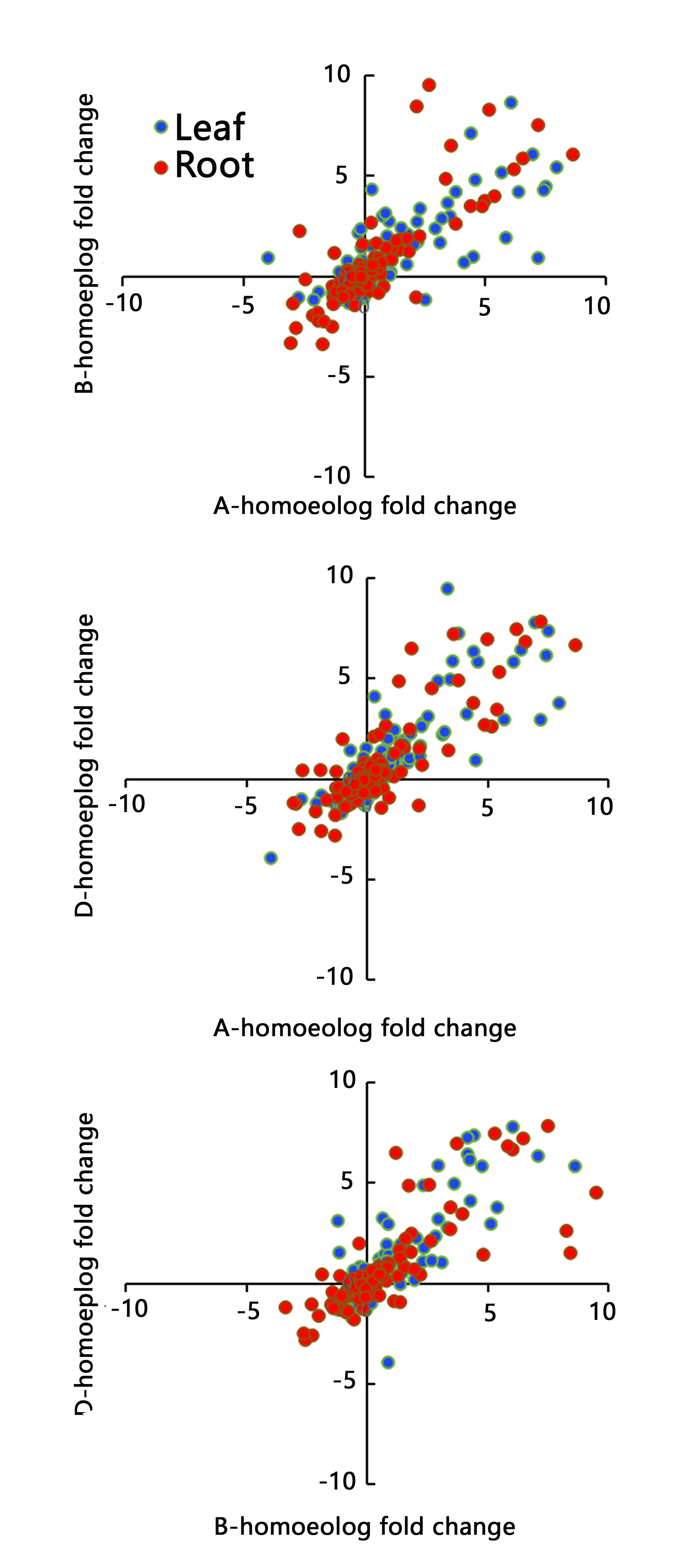

Supplement: Supplementary file 1 — Additional file 1: Figure S1. Kyoto Encyclopedia of Genes and Genomes (KEGG) enrichment of differentially expressed genes in allohexaploid wheat leaf. Top 20 KEGG pathways with adjusted P value<0.05 are displayed. Figure S2. Kyoto Encyclopedia of Genes and Genomes (KEGG) enrichment of differentially expressed genes in allohexaploid wheat root. KEGG pathways with adjusted P value<0.05 are displayed. Figure S3. Effects of long-term salinity stress on gene expression involved in wax biosynthesis. (a) Gene expression change was marked on the pathway of wax biosynthesis, and the red box indicates up-regulated expression under long-term salinity stress. The wax biosynthesis pathway diagram was adapted from the diagram of KEGG website. (b) Gene expression data involved in wax metabolism in wheat leaf under long-term salinity stress. FAR, alcohol-forming fatty acyl-CoA reductase. MAH1, midchain alkane hydroxylase; CYP96A15; WSD1, wax-ester synthase/diacylglycerol O-acyltransferase. Fold change = stress/control, Q value is the adjusted P value using the Benjamini-Hochberg method. Figure S4. Effects of long-term salinity stress on expression of two ferredoxin-NADP+ reductase (petH) genes in wheat leaves. Figure S5. Relationships among A, B and D homeologs in terms of expression fold change (stress/control) for all salinity-tolerant triads. Figure S6. Expression fold change (stress/control) of A, B and D homeologs of 15 typical salinity-tolerant triads. * indicates significant difference (adjusted P value < 0.05 and |log2fold change| ≥ 1). [file 12870_2020_2423_MOESM1_ESM.zip › Figure S5 fold change of homoelog of salinity gene.tif]

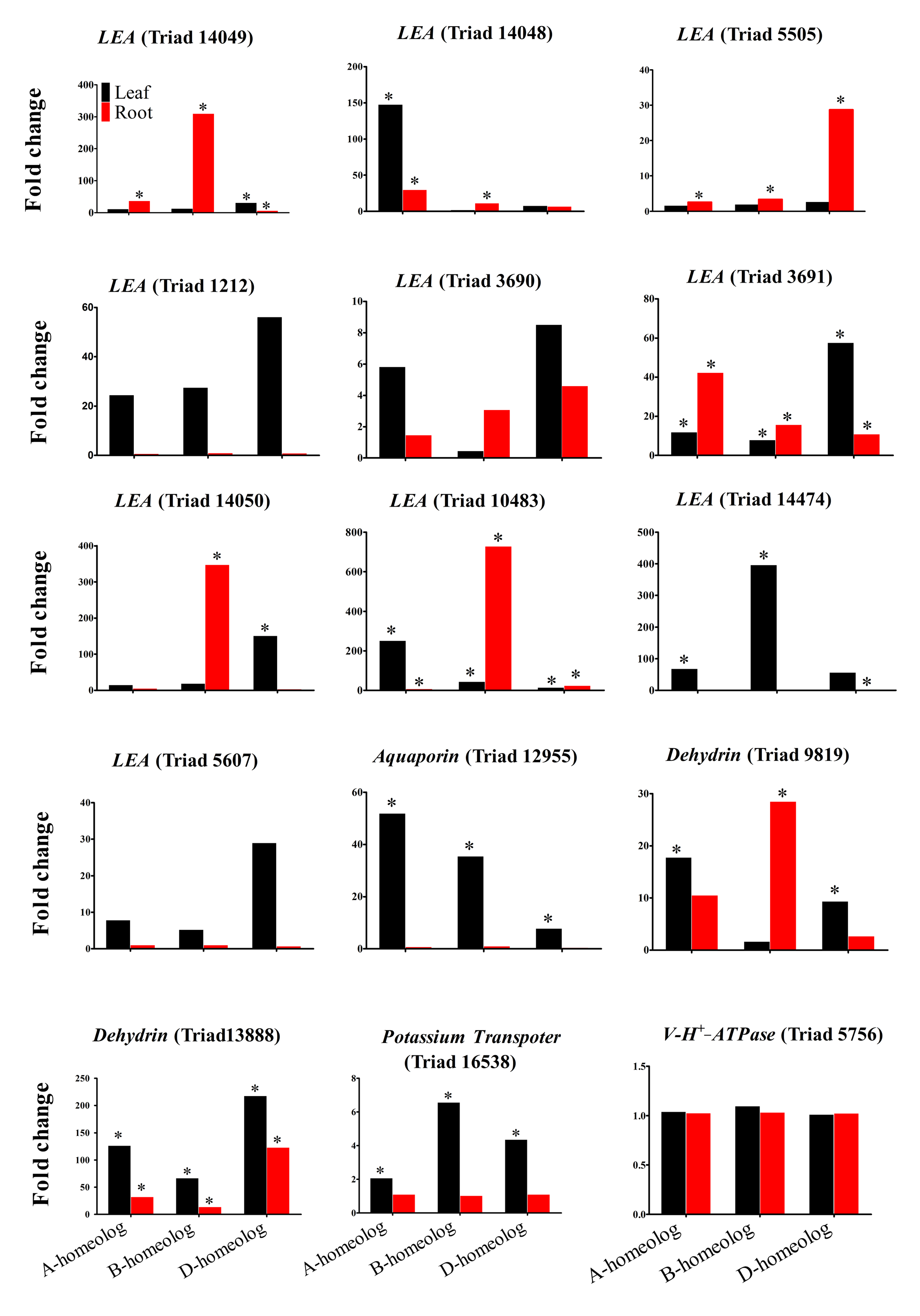

Supplement: Supplementary file 1 — Additional file 1: Figure S1. Kyoto Encyclopedia of Genes and Genomes (KEGG) enrichment of differentially expressed genes in allohexaploid wheat leaf. Top 20 KEGG pathways with adjusted P value<0.05 are displayed. Figure S2. Kyoto Encyclopedia of Genes and Genomes (KEGG) enrichment of differentially expressed genes in allohexaploid wheat root. KEGG pathways with adjusted P value<0.05 are displayed. Figure S3. Effects of long-term salinity stress on gene expression involved in wax biosynthesis. (a) Gene expression change was marked on the pathway of wax biosynthesis, and the red box indicates up-regulated expression under long-term salinity stress. The wax biosynthesis pathway diagram was adapted from the diagram of KEGG website. (b) Gene expression data involved in wax metabolism in wheat leaf under long-term salinity stress. FAR, alcohol-forming fatty acyl-CoA reductase. MAH1, midchain alkane hydroxylase; CYP96A15; WSD1, wax-ester synthase/diacylglycerol O-acyltransferase. Fold change = stress/control, Q value is the adjusted P value using the Benjamini-Hochberg method. Figure S4. Effects of long-term salinity stress on expression of two ferredoxin-NADP+ reductase (petH) genes in wheat leaves. Figure S5. Relationships among A, B and D homeologs in terms of expression fold change (stress/control) for all salinity-tolerant triads. Figure S6. Expression fold change (stress/control) of A, B and D homeologs of 15 typical salinity-tolerant triads. * indicates significant difference (adjusted P value < 0.05 and |log2fold change| ≥ 1). [file 12870_2020_2423_MOESM1_ESM.zip › Figure S6 fold change of 15 typic salinity triads.tif]
